# Prospective study of vaginal samples for the detection of *Gardnerella vaginalis* and *G. swidsinskii/leopoldii* using Gardnerella agar in CO_2_ and anaerobic atmospheres

**DOI:** 10.1128/spectrum.03668-25

**Published:** 2026-05-06

**Authors:** Irena Grmek Košnik, Ana Ribnikar, Ema Ogrizek, Jelena Kovačević

**Affiliations:** 1 National Laboratory for Health, Environment and Food, Kranj, Slovenia; University of Heidelberg, Heidelberg, Germany

**Keywords:** bacterial vaginosis, *Gardnerella vaginalis*, *G. leopoldii/swidsinskii*, cultivation, diagnostics

## Abstract

*Gardnerella vaginalis* is a key bacterium in the etiology of bacterial vaginosis (BV) and contributes to biofilm formation. *Gardnerella* is a facultative anaerobic bacterium, meaning it can grow both in the presence of oxygen and under anaerobic conditions. We conducted a prospective study in 2025, including 160 consecutive vaginal samples from women submitted for pathogenic bacteria. Clinical information on the referral form indicated in 87 women (54.4%), from which colpitis in 64 cases (73.6%). There were 43 pregnant women (26.9%), with an average age of 30.7 years. Each vaginal sample was cultured on Gardnerella agar and incubated in both CO_2_ and anaerobic atmospheres. The culture plates were examined after 24 and 48 h of incubation, independently of other results. In cases where suspicious colonies with beta-hemolysis were observed, bacterial identification was performed using MALDI-TOF mass spectrometry (Bruker). Gram-stained smears were evaluated according to the Nugent score. The bacteria *Gardnerella vaginalis* and *Gardnerella leopoldii/swidsinskii* were detected in 46 (28.8%) samples. The sensitivity of *G. vaginalis* and *G. leopoldii/swidsinskii* cultivation on Gardnerella agar in a CO_2_ atmosphere was 67.4% compared to anaerobic cultivation. Anaerobic cultivation on Gardnerella agar proved to be a superior method for the isolation of *G. vaginalis* and *G. leopoldii/swidsinskii* compared to cultivation in a CO_2_ atmosphere. The diagnosis of BV in clinical practice relies on standard Amsel’s criteria, gram-stained vaginal smears evaluated using the Nugent scoring system or Ison-Hay criteria, and the isolation of *G. vaginalis* and *G. leopoldii/swidsinskii*.

**IMPORTANCE:**

Bacterial vaginosis (BV) is the most common vaginal infection among women of reproductive age and is associated with significant adverse outcomes, including preterm birth, spontaneous abortion, and increased susceptibility to sexually transmitted infections. Accurate and timely microbiological diagnosis is therefore clinically important. Our study demonstrates that anaerobic incubation on selective Gardnerella agar improves the detection of *Gardnerella vaginalis* and *Gardnerella leopoldii*/*swidsinskii* compared with incubation in a CO_2_-enriched atmosphere. Under anaerobic conditions, colonies were larger, hemolysis was more pronounced, and overall sensitivity was higher. Although BV diagnosis in clinical practice primarily relies on Amsel’s criteria and Nugent scoring, optimized culture conditions provide valuable complementary microbiological evidence. These findings contribute to the optimization of laboratory procedures and may enhance the reliability of routine diagnostic workflows in women with suspected BV.

## INTRODUCTION

Bacterial vaginosis (BV) is the most prevalent vaginal infection among women of reproductive age and is linked to several significant adverse health outcomes. Its prevalence in the general population is high worldwide, estimated to range from 23% to 29% across different regions (Europe and Central Asia, 23%; East Asia and the Pacific, 24%; Latin America and the Caribbean, 24%; the Middle East and North Africa, 25%; sub-Saharan Africa, 25%; North America, 27%; and South Asia, 29%). In North America, prevalence differs notably by race and ethnicity, with higher rates observed among Black and Hispanic women (33% and 31%, respectively) compared with other groups (White, 23%; Asian, 11%; *P* < 0.01) (1). BV can be asymptomatic or present with symptoms such as pruritus, foul-smelling vaginal discharge, and burning during urination (2). It has been associated with various adverse health outcomes, including infertility, preterm birth, spontaneous abortion, increased susceptibility to HIV and other sexually transmitted infections (e.g., *Chlamydia trachomatis*, *Neisseria gonorrhoeae*, HPV, HSV-2, *Trichomonas vaginalis*, and *Mycoplasma genitalium*), as well as pelvic inflammatory disease and endometritis (2, 3). The condition is more common among menstruating women and those with copper intrauterine devices (2).

Normal vaginal microbiota predominantly consists of lactobacilli, including *Lactobacillus crispatus*, *Lactobacillus gasseri*, *Lactobacillus iners*, and *Lactobacillus jensenii*. These bacteria play a protective role by producing antimicrobial compounds, such as lactic acid and hydrogen peroxide, which help maintain a low vaginal pH. However, *L. iners* can be present in both BV-positive and BV-negative women (4, 5).

BV is characterized by polymicrobial dysbiosis, involving a 100–1,000-fold increase in facultative (*Gardnerella* spp.) and strictly anaerobic bacteria (*Atopobium vaginae*, *Mobiluncus* spp., *Bifidobacterium* spp., *Megasphaera* types, *Sneathia sanguinegens*, *Porphyromonas asaccharolytica*, *Prevotella bivia*, *Leptotrichia amnionii*, and *Peptoniphilus* spp.) (6). *Atopobium vaginae* was recently reclassified as *Fannyhessea vaginae* (7). BV represents the acquisition of a diverse community of anaerobic and facultative bacteria and a reduction in lactobacilli; they have been termed BV-associated bacteria, serving as highly specific indicators of BV (5). *Gardnerella vaginalis* is considered a principal pathogen in BV, although the specific contributions of the various *Gardnerella* species are not yet fully understood. Among them, *G. vaginalis* was the most frequently detected species (36.0%). All examined *Gardnerella* species were commonly identified in women with (asymptomatic) BV, with prevalence ranging from 50.9% to 57.9%. However, univariate analysis showed no significant associations between any *Gardnerella* species and the clinical symptoms of BV. While all species appeared to play a role in BV, none were linked to its most prominent clinical manifestations (8).

MALDI-TOF analysis and biochemical studies led to the classification of three new species: *Gardnerella piotii* (sialidase-positive, β-galactosidase-negative), *Gardnerella swidsinskii* (sialidase-negative, β-galactosidase-negative), and *Gardnerella leopoldii* (sialidase-negative, β-galactosidase-negative) (9). Only *G. vaginalis* produces β-galactosidase (10). *G. vaginalis* is detected in 98%–100% of BV cases but can also be isolated from 55% of BV-negative women (11).

The clinical diagnosis is based on Amsel’s criteria, with at least three out of four criteria required to be met: a homogeneous whitish-gray vaginal discharge, vaginal pH > 4.5, a positive whiff-amine test (a fishy odor develops upon the addition of 10% potassium hydroxide to the vaginal discharge) and presence of >20% clue cells (desquamated squamous epithelial cells covered with small coccobacilli, giving them a stippled, granular appearance; the borders of the epithelial cells are not clearly defined due to the large number of bacteria and cellular disintegration). To detect clue cells, a drop of vaginal discharge should be mixed with normal saline on a microscope slide, covered with a coverslip, and examined under a microscope at 400× magnification (12, 13).

BV is diagnosed microbiologically by Gram staining of vaginal smears. There are traditionally two methods of scoring vaginal smears—the Nugent and Ison-Hay. The first was described by Spiegel et al. and modified by Nugent et al. (14, 15). This system quantifies different bacterial morphotypes, which requires an experienced microscopist and is subjective in interpretation. To determine the Nugent score, vaginal swabs are examined for the presence of gram-positive bacilli (*Lactobacillus* morphotypes; count 0–4), small gram-variable genera (*Gardnerella* morphotypes; count 0–4), and curved gram-variable genera (*Mobiluncus* morphotypes; count 0–2), with a score ranging from 0 to 10. A score of 0–3 is defined as optimal vaginal microbiota, 4–6 as intermediate vaginal microbiota, and 7–10 as BV. The Nugent score is the reference standard laboratory method for diagnosing BV, i.e., the gold standard (13, 16).

The Nugent score is the reference laboratory method for diagnosing BV and is widely regarded as the gold standard (13, 16). Due to the complexity of this method, a simplified vaginal Gram stain interpretation system was later introduced by Ison and Hay in 2002. In this system, the vaginal microbiota is categorized into three groups—normal, intermediate, or BV—based on the relative abundance of *Lactobacillus* morphotypes (many, equal numbers, or few) compared with *Gardnerella* morphotypes (few, equal numbers, or many). These categories correspond to grade I (normal), grade II (intermediate), and grade III (BV) (17). Formerly classified as a single species, *Gardnerella vaginalis*, the *Gardnerella* genus is now known to comprise at least 13 distinct genomospecies. Of these, six have been formally named: *Gardnerella vaginalis*, *Gardnerella piotii*, *Gardnerella swidsinskii*, *Gardnerella leopoldii*, *Gardnerella pickettii*, and *Gardnerella greenwoodii*. Previous studies have also demonstrated that the vagina can be colonized by multiple *Gardnerella* species simultaneously, underscoring the importance of further investigating the prevalence and specific roles of individual species within the *Gardnerella* genus (18, 19).

In patients with BV, higher concentrations and prevalence of each *Gardnerella* species group were found. Among BV-positive patients, 91.1% had three or more *Gardnerella* species groups detected, compared with 32.0% of BV-negative participants (*P* < 0.0001). These findings indicate that BV is associated with a state of increased *Gardnerella* species diversity. However, no single *Gardnerella* species group was identified as a specific marker for BV (19). Culture of *Gardnerella* spp. is highly sensitive but not specific for BV, since these bacteria may also be present in women without BV. Nevertheless, *G. vaginalis* is central to BV pathogenesis, and our study showed that anaerobic cultivation on selective agar improves detection compared to CO_2_ conditions. Thus, while Amsel’s criteria and Nugent scoring remain the gold standards, *Gardnerella* culture provides useful complementary evidence.

In our study, we aimed to determine the significance of *Gardnerella* spp. isolation by comparing the culture with the presence of clue cells and Nugent evaluation. We also aimed to determine the optimal conditions for the growth and detection of *Gardnerella* spp. in the microbiology laboratory. In our study, the CO_2_ atmosphere corresponded to incubation in 5% CO_2_ using a standard CO_2_ incubator. These conditions are not strictly microaerophilic but represent capnophilic incubation with ambient oxygen concentration (approximately 20%–21% O_2_). The oxygen concentration is the principal differentiating factor between the two incubation systems. We did not find any articles in the literature that compared the cultivation of *Gardnerella* under anaerobic conditions and in an aerobic atmosphere.

## MATERIALS AND METHODS

### Study design and setting

This prospective observational study was conducted at the Department of Medical Microbiology, Kranj, National Laboratory for Health, Environment and Food (NLZOH), Slovenia, during two study periods: 17 April to 14 May 2025 and 25 August to 4 September 2025.

### Study population and specimens

A total of 160 consecutive, non-selected vaginal swabs submitted for routine microbiological investigation of pathogenic bacteria were included. Samples originated from

- Primary Health Care in Gorenjska
- Ljubljana Community Health Center
- Jesenice General Hospital
- Hospital for Gynecology and Obstetrics Kranj

Only clearly documented clinical diagnoses provided on referral forms were included in the analysis.

### Sample processing and culture conditions

All vaginal swabs were transported in Amies CLR, CE IVD transport medium (MEUS Srl, Italy) and processed within 4–24 h after collection. Samples were stored and transported at room temperature according to the manufacturer’s instructions and routine laboratory practice.

Each specimen was inoculated into 5 mL tryptic soy broth to standardize inoculum concentration. Subsequently, 10 µL of broth was plated onto

- Blood agar (KAO-CO_2_)
- Chocolate agar (CA-CO_2_)
- Gardnerella agar incubated in 5% CO_2_ (GAR-CO_2_)
- Gardnerella agar incubated under anaerobic conditions (GARana)

Anaerobic conditions (<0.1% O_2_ and >15% CO_2_) were generated using GENbox anaer (bioMérieux, France).

All plates were incubated at 35–37°C and examined after 24 and 48 h independently of other laboratory findings. Routine cultivation in our laboratory lasts a minimum of 48 h.

Anaerobic cultivation on Gardnerella agar was predefined as the reference method for comparative analysis.

### Colony assessment and bacterial identification

Colonies suspected of belonging to *Gardnerella* spp. were defined as small gray colonies demonstrating hemolysis on human blood-supplemented Gardnerella agar.

Identification was performed using MALDI-TOF mass spectrometry (Bruker). Only identifications with a score >2.0 were accepted.

Due to database limitations, MALDI-TOF could not differentiate

- *G. leopoldii* from *G. swidsinskii* (reported as *G. leopoldii/swidsinskii*)
- *G. vaginalis* from *G. piotii* (reported as *G. vaginalis*)

### Microscopic evaluation

Gram-stained vaginal smears were evaluated according to the Nugent scoring system.

Clue cells were assessed in native preparations using light microscopy (1,000× magnification). The presence of clue cells was used as the microbiological reference (“gold standard”) for comparison with culture results.

### Outcome measures

Primary outcome:

- Detection rate of *Gardnerella* spp. under CO_2_ vs anaerobic cultivation

Secondary outcomes:

- Agreement between culture results and
  - Presence of clue cells
  - Nugent score (7–10 considered positive for BV)
- Comparison of hemolysis patterns and colony morphology under different atmospheric conditions

### Statistical analysis

Data were analyzed using Microsoft Excel 2019 and SPSS version 28.0 (IBM Corp.).

Descriptive statistics included means and proportions. Differences between categorical variables were analyzed using

- Pearson’s chi-squared test (*χ*²)
- Fisher’s exact test with Bonferroni correction
- McNemar exact two-sided test (paired comparisons)
- Student’s *t*-test and ANOVA where appropriate

A *P* value <0.05 was considered statistically significant.

## RESULTS

During the specified period, 160 consecutive vaginal samples were received from women with a mean age of 39.14 years (range: 19–87 years). Clinical data were obtained from routine referral forms written by physicians. Only clearly stated diagnoses on referral forms were included. The information on the referral form indicated in 87 women (54.4%) from which colpitis in 64 cases (73.6%), including recurrent colpitis in 11 cases (12.6%). Discharge was noted in seven cases, bacterial vaginosis in two cases, status post-pelvic inflammation in one case. There were 43 pregnant women (26.9%) with an average age of 30.7 years. Gestational age was 23.9 weeks (range: 6–36 weeks).

Clue cells were observed in native specimens in 52 (32.5%) cases, with *Gardnerella* detected in 46 cases and *Prevotella bivia* in 2 cases. In five cases, we detected clue cells but did not isolate *Gardnerella* spp*. Gardnerella vaginalis* and *Gardnerella leopoldii/swidsinskii* were detected in 46 (28.8%) samples. Among these, clinical diagnosis by the physician was recorded in 27 of the 46 confirmed cases (58.7%). Specifically, *G. vaginalis* alone was identified in 24.4% of samples, *G. leopoldii/swidsinskii* alone in 4.4%, both *G. vaginalis* and *G. leopoldii/swidsinskii* in 3.1%. Other detected pathogens included *Streptococcus agalactiae* in 4.4%, *Escherichia coli* in 3.8%, and *Prevotella bivia* in 1.3%; *Haemophilus influenzae*, *Klebsiella pneumoniae*, and *Proteus mirabilis* were each detected in <1% of samples.

Yeast fungi were isolated in 21.3% of cases: *Candida albicans* (18.1%), *Candida glabrata* (1.3%), *Candida dubliniensis* (1.3%), and *Saccharomyces cerevisiae* (0.6%).

### Comparison of cultivation conditions

**Fig 1:**
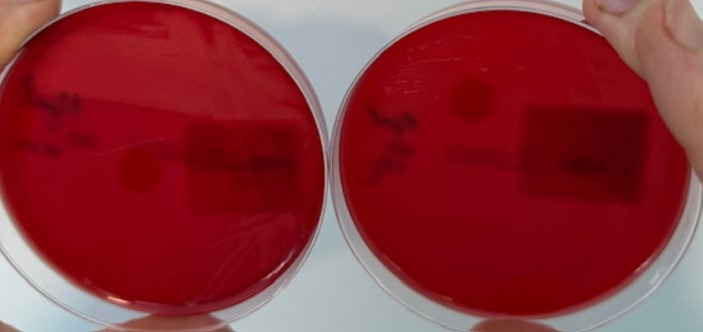
Detection of *G. vaginalis* was superior under anaerobic conditions after both 24 and 48 h of incubation, as colonies were larger (2 mm after 24 h and 3 mm after 48 h) and showed beta-hemolysis already after 24 h.

**Fig 2:**
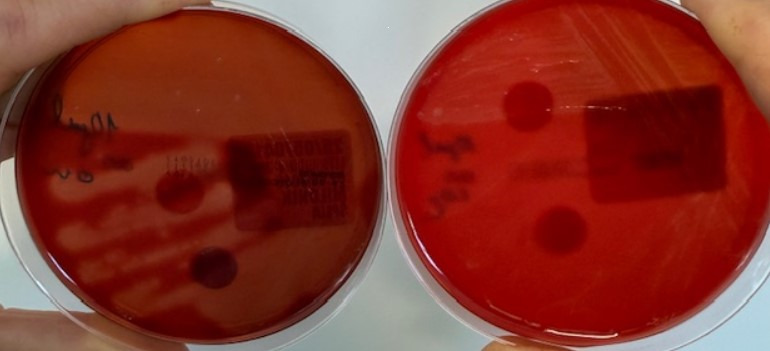
In contrast, on Gardnerella agar under CO_2_ conditions (GAR-CO_2_), alpha-hemolysis was present at 24 h, whereas beta-hemolysis emerged only after 48 h.

Detection of *G. vaginalis* was superior under anaerobic conditions after both 24 and 48 h of incubation, as colonies were larger (2 mm after 24 h and 3 mm after 48 h) and showed beta-hemolysis already after 24 h (Fig. 1). In contrast, on Gardnerella agar under CO_2_ conditions (GAR-CO_2_), alpha-hemolysis was present at 24 h, whereas beta-hemolysis emerged only after 48 h (Fig. 2).

In our study, the presence of clue cells was considered the gold standard against which the yield of various cultures was compared. We also evaluated the agreement between bacterial isolation (*G. vaginalis* and *G. leopoldii/swidsinskii*), clue cells, and Nugent scoring.

A positive Nugent score (7–10) was detected in *Gardnerella* spp. in 54.4%, in *G. vaginalis* in 52.9%, and in *G. leopoldii/swidsinskii* in 42.9%.

When observing the preparations where we isolated *G. leopoldii/swidsinskii*, we noticed that the bacilli are slightly larger and form clusters that are often not on epithelial cells, so we cannot define these as clue cells.

**Fig 3:**
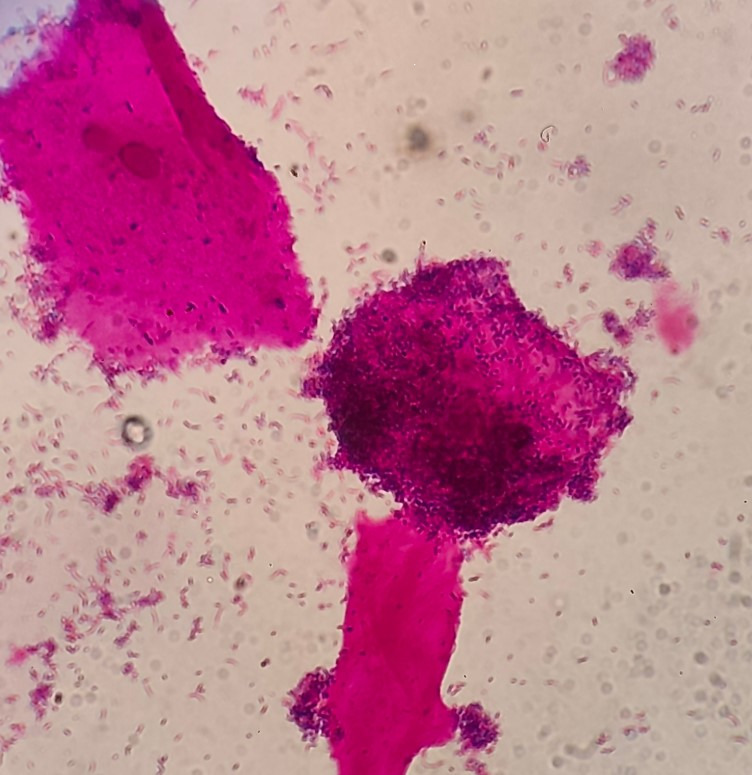
Clue cells are vaginal epithelial cells that are characteristically covered with a dense layer of adherent bacteria, giving their borders a fuzzy or stippled appearance under the microscope.

**Fig 4:**
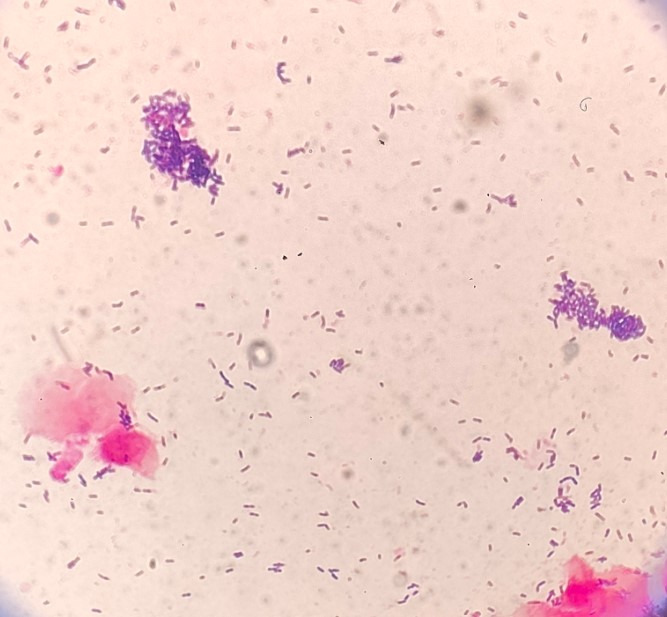
Clusters that are not attached to epithelial cells in *G. leopoldii/swidsinskii*.

Figure 3 shows the comparison of clue cells, and Fig. 4 shows the clusters that are not attached to epithelial cells. As a result, the evaluation of the average Nugent score in *G. leopoldii/swidsinskii* is also worse.

The sensitivity of all *Gardnerella* spp. cultivation on Gardnerella agar in a CO_2_ atmosphere was 67.4% (*Gardnerella vaginalis* was 73.5% and *G. leopoldii/swidsinskii* was 14.3%) when compared to anaerobic cultivation, with a specificity of 100% (Table 1).

**TABLE 1:** Results of the comparison of cultivation conditions using the same samples^*c*^

	Positive clue cells	Positive Nugent score (7–10)	CO_2_ conditions 24 h	CO_2_ conditions 48 h	Anaerobic conditions 24 h	Anaerobic conditions 48 h	*P* value^*b*^, CO_2_ conditions 24 h vs CO_2_ conditions 48 h	*P* value^*b*^, anaerobic conditions 24 h vs anaerobic conditions 48 h	*P* value^*b*^, CO_2_ conditions 48 h vs anaerobic conditions 48 h
For all *Gardnerella* spp.	43/46 (91.30%)	25/46 (54.35)	8/46 (17.39%)	31/46 (67.39%)	29/46 (63.04%)	46/46 (100%)	<0.01	<0.01	<0.01
For *G. vaginalis*	31/34 (91.18%)	18/34 (52.94)	4/34 (11.76%)	25/34 (73.52%)	23/34 (67.65%)	34/34 (100%)	<0.01	<0.01	<0.01
For *G. leopoldii/swidsinskii*	1/7 (85.71%)	3/7 (42.86)	0/7 (0%)	1/7 (14.29%)	4/7 (14.29%)	7/7 (100%)	NS^*d*^	NS	0.03
*P* value^*a*^, *G. vaginalis* vs *G. leopoldii/swidsinskii*	<0.01	NS	NS	0.03	NS	NS			

^*a*^ Two-sided Fisher’s exact test with Bonferroni correction.

^*b*^ McNemar exact two-sided test.

^*c*^ Only cases where *Gardnerella* grew on any plate were included in the analysis.

^*d*^ “NS” indicates not significant.

## DISCUSSION

*Gardnerella vaginalis* is a key bacterium in the etiology of BV. It belongs to the facultative anaerobic pleomorphic coccobacilli of the *Bifidobacteriaceae* family. In the presence of 5% CO_2_, the bacterium grows on chocolate agar as white-gray colonies and on Gardnerella agar with the addition of human blood, on which it forms beta-hemolysis. It also grows under microaerophilic conditions (10, 20). Isolation of *G. vaginalis* from clinical specimens has a high sensitivity (100%) but a low specificity (49%) for the diagnosis of BV (20).

Our study contributes to the optimization of laboratory methodologies by demonstrating that anaerobic cultivation provides a more reliable and effective approach for the isolation of *Gardnerella vaginalis* and *Gardnerella leopoldii/swidsinskii* compared to cultivation in a CO_2_-enriched atmosphere. Under anaerobic conditions, *Gardnerella* colonies grown on selective Gardnerella agar exhibit pronounced beta-hemolysis within 24 h and display approximately double the colony thickness, indicating enhanced growth characteristics (Fig. 1).

*Gardnerella* spp. is often found even in the absence of BV in both sexually experienced and inexperienced women (20). *G. vaginalis* is the main bacterium that produces biofilm, although by itself it is not sufficient for the formation of BV (5, 13). It is important for the initial formation of a biofilm, which is strengthened and expanded by the synergistic effects of metabolites from other participating bacteria in the community (*Prevotella bivia*, *Atopobium vaginae*, and *Fusobacterium* spp.). Virulent strains of *G. vaginalis* displace lactobacilli and initiate biofilm formation. *G. vaginalis* initiates proteolysis, producing amino acids that enhance the growth of *Prevotella bivia*, which in turn joins *G. vaginalis* in forming the biofilm. *G. vaginalis* and *P. bivia* produce sialidase, which causes the destruction of the mucus layer on the vaginal epithelium (21). *G. vaginalis* produces cytolysins, the most studied of which is vaginolysin, which activates protein kinase and causes lysis of vaginal epithelial cells (20). Other bacterial species, such as *F. vaginae* and *Fusobacterium* spp., stimulate and strengthen the biofilm formed by *G. vaginalis. G. vaginalis* is the dominant bacterium in biofilms, followed by *F. vaginae*, which is present in 80% of biofilms and contributes 40% of the biofilm mass. The simultaneous presence of *G. vaginalis* and *F. vaginae* in the vaginal biofilm is therefore important for the diagnosis of BV. *F. vaginae* was detected by PCR in 96% of women with BV and in 12%–19% of women without BV (22).

In the current study, *G. vaginalis* and *G. leopoldii/swidsinskii* were identified in 28.75% of the analyzed specimens. In a population of symptomatic women with vaginitis in Greece, 40.4% were *Gardnerella vaginalis*, 42.5% *Candida* spp., and 8.1% *Trichomonas vaginalis*. Less usual isolates were *Escherichia coli*, *Streptococcus agalactiae*, *Enterococcus* spp., *Streptococcus viridans*, *Staphylococcus epidermidis*, *Peptostreptococcus* spp., and *Staphylococcus saprophyticus* (23).

However, certain *G. vaginalis* strains proved to be strictly anaerobic (24). *G. vaginalis* is associated with ammonia odor, pH increase, and the presence of clue cells in Amsel standards (25), and also participates in the formation of vaginal biofilms. In fact, as an opportunistic pathogen, colonization by *Gardnerella* spp. does not always lead to BV. The causes of this condition need resolution so that the role of *Gardnerella* in BV pathogenesis can be properly understood. It is not only present in the vagina of BV patients but also can be detected in the vagina of non-BV patients (26). Some studies have shown that some lineages of *Gardnerella* may have enhanced pathogenicity, while others may be symbiotic strains that lack many of these pathogenic traits (27). For example, studies have shown that the vaginolysin expression of *Gardnerella vaginalis* isolated from the vagina of BV patients is higher than that of non-BV patients. Advances in molecular biology have led to a new understanding of the diversity of *Gardnerella*, leading to a revised description of *Gardnerella vaginalis. Gardnerella* spp. produces many toxic factors, such as vaginolysin and sialidase, which have been the most extensively studied virulence factors. Vaginolysin is a cholesterol-dependent cytolysin that can cause the dissolution of human erythrocytes, epithelial cells, and polymorphonuclear leukocytes (28).

Sialidase increases the cytotoxic activity of *Gardnerella* by degrading various key vaginal mucosal protective factors, such as mucin, and promotes exfoliation and abscission of vaginal epithelial cells (28). Given the important role of *Gardnerella* in the pathogenesis of BV, there remains a lot of work to be done on the basic biology and metabolic characteristics of *Gardnerella* as well as the roles of different *Gardnerella* strains in the pathogenesis of BV.

A study on bacterial interactions in bacterial vaginosis showed that anaerobic conditions are crucial for the growth and survival of *Gardnerella vaginalis*, especially when co-cultivated with other bacteria such as *Atopobium vaginae* (21). The anaerobic environment supports *G. vaginalis* biofilm formation, which is essential for its pathogenicity in the vaginal ecosystem. Overall, the anaerobic approach to cultivation is increasingly recognized as a more accurate method for studying and isolating *G. vaginalis*, as it better reflects its natural environment compared to aerobic methods (28).

In our study, anaerobic cultivation on Gardnerella agar was used as the reference method, against which results obtained under CO_2_ conditions were compared. This allowed us to validate the sensitivity and specificity of *Gardnerella* isolation across different cultivation environments. In addition, we assessed the agreement of bacterial isolation with clue cells and the Nugent score, thereby aligning our results with established diagnostic standards. While Amsel’s clinical criteria remain central to BV diagnosis in clinical practice, our findings highlight that cultivation methods can provide complementary microbiological evidence, especially in cases with inconclusive or borderline clinical findings. To strengthen these observations, we now include photographic documentation of representative *Gardnerella* colonies, demonstrating differences in colony morphology and hemolysis patterns under anaerobic vs CO_2_ conditions. These images support our conclusion that anaerobic cultivation provides superior conditions for the reliable isolation of *Gardnerella* species (Fig. 1 and 2).

Molecular methods, particularly quantitative PCR targeting *Gardnerella vaginalis* and other BV-associated bacteria, provide high sensitivity and specificity and are increasingly considered reference tools for BV diagnosis. However, culture-based methods remain relevant in routine clinical microbiology for several reasons. First, molecular diagnostics may not be universally available, particularly in smaller laboratories or resource-limited settings. Second, PCR detects bacterial DNA but does not provide viable isolates required for phenotypic characterization, antimicrobial susceptibility testing, or epidemiological strain comparison. Third, culture enables the assessment of colony morphology, hemolysis patterns, and growth characteristics under different atmospheric conditions, which may be relevant for understanding pathogenic potential. Therefore, culture should not be viewed as a replacement for molecular diagnostics, but rather as a complementary tool, particularly when viable isolates are required.

Our study has several limitations. First, molecular methods were not performed, which would have allowed species-level confirmation beyond MALDI-TOF limitations (inability to distinguish between *G. leopoldii* and *G. swidsinskii*, or between *G. vaginalis* and *G. piotii*). Second, the study was based on 160 vaginal specimens, which we recognize as a modest sample size. Third, while demographic information was systematically collected, clinical referral data were often incomplete, since physicians inconsistently provided detailed clinical diagnoses. Only clearly written diagnoses were included, which limited the scope of clinical correlation. Finally, although we strived for independent evaluation of each cultivation, the comparative assessment of colony morphology and Nugent scoring may have introduced some bias. Despite these limitations, our results provide important laboratory insights and emphasize the need for closer collaboration with clinicians in future studies, which will enable us to link microbiological findings more systematically with clinical outcomes and treatment follow-up.

The present study demonstrated that anaerobic cultivation on Gardnerella agar is a more reliable and effective method for the isolation of *Gardnerella vaginalis* and *G. leopoldii/swidsinskii* compared to cultivation under CO_2_ conditions. Our findings showed higher sensitivity under anaerobic conditions, with clearer colony morphology and faster onset of hemolysis, supporting the importance of appropriate cultivation conditions for accurate microbiological diagnosis of bacterial vaginosis.

While Amsel’s criteria and Nugent scoring remain the cornerstones of BV diagnosis, our results underline the value of complementary cultivation methods in clinical microbiology, particularly in cases with inconclusive clinical or microscopic findings. The addition of photographic evidence in this revised version provides further support for the enhanced growth characteristics observed under anaerobic conditions.

In the future, we plan to establish closer collaboration with clinicians and involve patients in defining symptoms, which will allow us to systematically link microbiological findings with detailed clinical outcomes and treatment follow-up. Such integrated studies will provide deeper insights into the clinical significance of *Gardnerella* species and their role in bacterial vaginosis.

## Data Availability

The data sets presented in this article are not readily available. Requests to access the data sets should be directed to irena.grmek.kosnik@nlzoh.si.
